# Etiology Study of Acquired Developmental Defects of Enamel and Their Association with Dental Caries in Children between 3 and 19 Years Old from Dolj County, Romania

**DOI:** 10.3390/children9091386

**Published:** 2022-09-14

**Authors:** Mihai Popescu, Mihaela Ionescu, Monica Scrieciu, Sanda Mihaela Popescu, Răzvan Mercuţ, Marina Olimpia Amărăscu, Monica Mihaela Iacov Crăiţoiu, Daniela Lazăr, Veronica Mercuţ

**Affiliations:** 1 Department of Pedodontics, University of Medicine and Pharmacy of Craiova, 200349 Craiova, Romania; 2 Department of Medical Informatics and Biostatistics, University of Medicine and Pharmacy of Craiova, 200349 Craiova, Romania; 3 Department of Prosthodontics, University of Medicine and Pharmacy of Craiova, 200349 Craiova, Romania; 4 Department of Oral Rehabilitation, University of Medicine and Pharmacy of Craiova, 200349 Craiova, Romania; 5 Department of Plastic Surgery, University of Medicine and Pharmacy of Craiova, 200349 Craiova, Romania; 6 Mines ParisTech PSL, 92800 Paris, France

**Keywords:** developmental defects of enamel, odontogenesis, risk factors, caries, medication

## Abstract

Background: Developmental defects of enamel (DDE) are frequently encountered in primary and permanent teeth, yet their etiology is not completely known. Enamel hypoplasia is considered a predisposing factor for early caries. The objective of this study was the evaluation of several risk factors potentially causing DDE and the possible association between DDE and dental caries. Methods: This study was performed on a group of 213 rural children from Romania. It combined a thorough dental examination for all children, and a questionnaire filled in by their mothers, regarding the evolution of their pregnancy and the child’s health status in the first years of life. Results: There was no statistically significant association between DDE presence and data regarding the evolution of pregnancy, mothers’ health status or children’s conditions during early childhood. There was a significant association between the use of amoxicillin, ibuprofen, and cephalosporin during the period of formation of permanent teeth, and one environmental factor (water source), and the presence of DDE (Chi Square, *p* < 0.05). Also, DDEs were associated with the presence of caries (Fisher, *p* = 0.001). Conclusions: Children who consumed water from private wells and children who received medication during early childhood developed more enamel defects, presenting a higher risk of caries development.

## 1. Introduction

Quantitative and qualitative disorders of the hard tissue that covers the visible surface of primary and permanent teeth, are known as developmental defects of enamel (DDE) [1].

DDEs are not a recent discovery; they were initially described starting from the XVIIIth century, and the first studies on this subject date from 1746, and they were performed by Robert Bunon. He described the present of defects on the surface of unerupted teeth prelevated from dead children that suffered from rachitis, scurvy, measles, or smallpox [2]. Lately, DDEs have been extensively studied, due to their high prevalence and extensive changes that are produced at teeth level. In developed countries, the prevalence of DDEs in healthy children varies from 9% to 63% for permanent teeth, revealing an increasing trend [3].

Developmental defects of enamel (DDE) are frequently encountered in dental practice, both for primary and permanent teeth. However, there are still some missing data regarding their etiology, physiognomic modifications following changes in the morphology of the teeth, disorders of dental sensitivity and even changes in the occlusal function [3]. Moreover, enamel hypoplasia has been described as one of the predisposing factors for early caries and tooth wear [4].

Teeth development is genetically regulated but can be also sensitive to the action of systemic and/or local acquired risk factors [5]. The acquired DDE occur in the form as hypoplasia, which is a quantitative defect or hypomineralization (qualitative defect), in direct relation to the moment when the disturbance takes place [6]. Therefore, hypoplasia appears when the risk factors act during the secretory phase of amelogenesis, while hypomineralizations are caused by the aggressions occurring during the maturation stage of the tooth enamel [7]. Primary teeth are affected by DDE when the risk factors occur during pregnancy and in the first year of the child’s life, while permanent teeth defects are caused by disruptions that act during the first 7 years of the child’s life [8]. The type and appearance of the lesion do not depend on the type of the causative factor, but on the moment of its action, its duration and intensity.

There are many prenatal, perinatal, and postnatal etiological factors considered responsible for the development of DDE [9]. The term “risk factors” is more appropriate than “etiological factors” because the etiology of DDE is still ambiguous, with a multitude of factors having the potential to affect ameloblasts during odontogenesis and to cause these dental structural defects. That is why the etiology of DDE requires more studies.

On the other hand, teeth affected by DDE have high sensitivity due to rapid wear, with exposure to the dentin layer [10]. Moreover, children avoid eating on the side containing those teeth, so bacterial plaque biofilm tends to accumulate on the entire dental arch, thus even teeth without DDE may develop caries [11].

Hypomineralized enamel dissolves easily in acidic environment, so the affected teeth are prone to develop carious processes. It is considered that the defective enamel, which is uneven and retentive, is subject to higher bacterial plaque biofilm accumulation, also leading to developing caries on teeth without DDE. Thus, Americano et al. found in a systematic review a tight connection between dental caries and DDE, the affected children having 2- or 4-times higher chances to develop caries on any tooth than children from the control group [12]. This association between hypoplasia, opacities and dental caries is possible, but it remains unclear, additional research is needed in this regard [13,14].

DDE treatment represent a challenge for the dentist, as dental sensitivity and pain are difficult to withstand by the child patient. Besides these drawbacks, for teeth with DDE, local anesthesia is difficult to set in and most of the times, because of altered nerve potential, the restoration process fails [15].

In this context, preventive attitude must come first, and it must be oriented towards the identification and potential removal of etiological factors involved in DDE formation, and towards a correct diagnosis, to allow early and preferably non-invasive treatment. Therefore, the objective of the present study was to evaluate the risk factors potentially causing DDE and the possible association between DDE and dental caries on a group of rural children from Dolj County, Romania, aged from 3 to 19 years.

## 2. Materials and Methods

This research study is a continuation of a previous study carried out in January–February 2020, on 213 children aged 3–19, from Poiana Mare, Dolj county, Romania, enrolled at the “George Ștefan Marincu” High School, in which, based on the clinical examination, a prevalence of DDE was established of 11.27% [16].

It is a transversal statistical study which initially established the prevalence of DDE among children, based on clinical examination, and aims to identify the associated risk factors, using a questionnaire filled in by their mothers. All children who accepted to participate in this study and whose parents filled in the informed consent form, were included in the study group. Children with physical or mental disabilities, severe diseases in their past, or with braces were excluded from this study. Moreover, teeth with restorations covering more than 2/3 of the entire surface were also excluded.

The examination was performed by one dentist, in the classroom, under natural light, by visualizing the oral cavity. The child was asked to sit on a chair, in front of the examiner. No instruments were inserted in their oral cavity. The evaluation included the clinical examination, and all data were recorded by another dentist, using an Excel document, with odontograms for both types of dentitions. DDE quantification was performed based on the modified DDE index, specific for screening studies (Table 1) [17].

Dental caries were diagnosed based on the lack of dental hard tissues (carious cavity) and the presence of altered dentin. This covered all carious lesions, varying from incipient loss of hard tissue associated with altered dentin, up to only root remains. The international caries detection and assessment system “ICDAS II criteria” [18] could not be used, as the conditions of the examination were not suitable.

For each participant, the following parameters were retained: age, gender, residence, individual tooth analysis (emphasizing the presence and type of DDE, and caries).

In addition, a form comprising questions related to the risk factors involved in acquired DDE in children, was left to be filled in by the children’s mothers. The form was structured according to the following sections:

- generic data regarding gender, age, place of birth and place of residence for each child included in the study group;
- data related to the mother’s health status: conditions or complications during pregnancy or at birth, medication during pregnancy;
- birth details: type of birth (term or premature), weight of the child at birth, breastfeeding duration;
- child’s alimentation;
- child’s medical history in the first 4 years of life: presence of fever, various conditions (pneumonia, asthma, otitis, chickenpox, etc.); immunization for DTP, chickenpox, polio, hepatitis, mumps or others; allergies to medication (antibiotics, anti-inflammatory medication, general analgesics, anesthetics), food or other allergies; lack of calcium (declared by the child’s mother, according to data recorded by their general practitioner);
- data regarding the medication given to the child, since birth (amoxicillin, penicillin, anti-inflammatory medication, fluor supplements, etc.);
- sources of water (private source, the village’s water network, bottled water);
- sources of fruits and vegetables (local production with potential use of pesticides or bought from markets and stores).

The study was approved by the Ethics Commission of the University of Medicine and Pharmacy of Craiova and required the permission of the parents of the examined children, who were asked to fill in an informed consent (no. 125/9 December 2019).

### Statistical Analysis

Microsoft Excel software was used to regroup all digital records, analyze, interpret the collected data, graphically display the findings, and apply various techniques and procedures that completed the descriptive analysis. Continuous variables were defined as absolute and relative frequencies (%), as well as average ± standard deviation. Results were statistically analyzed based on the Chi-square, Fisher Exact and Mann-Whitney U tests for group distributions, with Statistical Package for Social Sciences (SPSS), version 20 (IBM Corp., New York, NY, USA), considering *p* < 0.05 as statistically significant.

## 3. Results

The study group comprised 213 children aged 3–19 years, from Poiana Mare, a rural area in Dolj county (Romania). A total of 24 children were diagnosed with DDE. For all participants, a total of 5452 teeth were investigated, and the analysis revealed 49 teeth with DDE.

### 3.1. Form Analysis

In addition to the observations gathered by the dentists included in this study, the mothers of all 213 participants from the study group (all with rural residence) provided a form with all information stated in the previous section. Since 24 children were initially diagnosed with developmental enamel defects, their mothers were included in the DDE group, while the others formed the no-DDE group.

All mothers reported an overall good health status during their pregnancy. Only 7 mothers (3.29% of our study group) had various conditions, properly compensated while being pregnant (3 of them were anemic, 2 presented arterial hypertension, and 2 had thrombophilia). Regarding the administration of medication during pregnancy, 10.33% (representing 22 mothers) took medication, most often vitamins. Our analysis revealed thar 25% of mothers from the DDE group took medication, compared to 11.1% mothers from the no-DDE group. A total of 5.16% participants (representing 11 mothers) presented complications at birth, one third requiring a C-section. The other complications were membrane fissure, loss of amniotic liquid, pregnancy with risk of abortion and contractions, bleeding. There was no statistically significant association between DDE presence in childhood and data regarding the evolution of pregnancy (Table 2).

The section corresponding to data regarding newborns indicated that their birth weight varied from 900 g to 4800 g, with an average value of 3245 g. Babies delivered prematurely had weights between 900 g and 2400 g; according to this criterion, 16 newborns (representing 7.5% of the entire study group) were premature, and 3 of them were later diagnosed with DDE. There was no statistically significant difference between DDE and no-DDE groups in term of premature births or babies’ weight at birth (Table 1).

Approximately 90% of mothers breastfed their babies (193 mothers) after birth. During the 4 first months, 32 mothers combined breastfeeding with formula milk, and 123 after month 4, until 1 year. Only 13 mothers did not breastfeed at all. The global breastfeeding duration had an average of 10 months, with a minimum value of 1 month and a maximum of 3 years). There was no statistically significant difference between DDE and no-DDE groups in term of breastfeeding or milk formula used, nor in term of duration (Table 3).

Our form also gathered data about children’s evolution during their first 4 years of life. Among our study group, 39 children (18.31% of the entire group, 2 from DDE group, 37 from no-DDE group) presented various conditions during their first 4 years of life. The most common conditions during early childhood were the following: chickenpox (18.31%), pharyngitis (10.33%), otitis (5.63%), pneumonia (1.88%), asthma (1.41%), amygdalitis (1.41%). The following conditions were rarely present: bronchitis (0.47%), constipation (0.47%), type I diabetes (0.47%), D.S.V.-D.S.A. (0.47%), umbilical hernia (0.47%). Fever was present in 75.12% of cases. There were no statistically significant differences between DDE and no-DDE groups in term of medical conditions during the first 4 years of life (Table 4).

Around 36% of children were vaccinated with DTP, chickenpox, poliomyelitis, hepatitis, and mumps. Only few children were allergic to antibiotics, aspirin, various foods, local or general analgesics, and none from the DDE group.

Regarding the medication administered in the first years of life for all children included in the study group, the collected forms indicated the following administration status: amoxicillin (42.25%, representing 90 children), penicillin (10.80%, representing 23 children), ibuprofen (7.98%, representing 17 children), paracetamol (7.04%, representing 15 children), cephalosporins (5.63%, representing 12 children), other medication (4.69%, representing 10 children). Statistical differences between DDE and no-DDE groups were identified for amoxicillin, ibuprofen and cephalosporins (Table 5).

The final section of the form analyzed the following parameters: lack of calcium, water source, fruit/vegetable sources and use of pesticides. Almost a quarter of children presented a lack of calcium (48, representing 22.64% from the entire study group), only 4 were part of the DDE group, and 44 were part of the no-DDE group.

The sources of water and fruits/vegetables were also investigated, and the analysis revealed that 77.46% of all participants (168 children, 23 from DDE group, 145 from no-DDE group) drank water from private wells; while the rest drank water from controlled sources (like bottled water or tap water from the local water network). A Chi-square test revealed statistically significant differences between DDE and no-DDE groups (Table 6).

Also, 61 children (only 8 with DDE) consumed fruits and vegetable from their private garden (representing 28.64% from the entire study group), 10 children (4.69%) from local stores (none with DDE), and 142 children (66.67%) used both sources (16 with DDE). The use of pesticides was declared by 30 mothers (only 2 from DDE group), representing 14.08% from the entire study group. There were no statistically significant differences between DDE and no-DDE groups in term of lack of calcium, sources of fruits/vegetables or use of pesticides (Table 6).

### 3.2. Caries Analysis

From the total of 5452 analyzed teeth (both permanent and primary teeth), 493 had caries (with and without DDE). The distribution is detailed in Table 7.

Regarding the teeth with DDE, from the total of 49 teeth, 8 permanent teeth had caries, while 4 primary teeth had caries, leading to a total of 12 teeth with both DDE and caries (24.49% from all teeth with DDE). Similarly, 5403 teeth do not have DDE, but 481 teeth had caries (0.089% from all teeth without DDE): 221 permanent teeth and 260 primary teeth. A Fisher’s Exact test was run between teeth with and without DDE, and teeth with and without caries. There was a statistically significant association between DDE and caries, *p* = 0.001.

## 4. Discussion

DDE represents a subject of interest for specialists in the field of dentistry due to its high and constantly increasing prevalence, especially in developed countries [19,20], due to the disorders it produces at teeth level, and occlusal function [3], and due to therapeutic difficulties [4]. In this context, an effective management of DDE should be based on prevention, by controlling the factors involved in the production of this condition and stopping the evolution of the lesions at an early stage.

Each tooth grows following a well-defined sequence, at the end of which it reaches its morphological and functional maturity [11]. This tooth development process can be disrupted by nutritional (malnutrition, ricketiness) and non-nutritional risk factors (infections during childhood). Therefore, the dental status can reflect the socio-economic level of the child and the disturbances to which it was exposed pre, peri and postnatally [21].

The study showed that a small number of mothers suffered from specific conditions or used drugs during pregnancy, and no correlation could be established between these and DDE. In literature, there are data on several maternal conditions during pregnancy, which would cause DDE in the child’s dentition. Thus, through the transmission of syphilis at birth from mother to child, hypoplasias are generated at the level of the child’s incisors [22]. DDE can also develop in the child’s primary dentition, if the mother is affected during pregnancy by mumps, measles, rubella, chickenpox, or influenza [23].

Regarding the relationship between DDE and drug use in pregnancy, Hong reported in 2011 that amoxicillin is a risk factor for the occurrence of developmental dental defects [24]. Regarding complications at birth and complications during pregnancy, in the presented study they were recorded in 5.16% of mothers, but no correlation with DDE was established.

Similar results were reported by Allazzam in 2014, who found no association between DDE and birth complications [25]; while a study on 1511 children aged between 8 and 11 years, conducted by Koruyucu in 2018, showed a significant association between complications during pregnancy and the presence of hypomineralization at the level of the 1st permanent molar and permanent incisors [26]. The difference in results may be explained by the fact that the group examined in the present study was much smaller and the data provided by mothers was subject to a margin of error and a degree of subjectivity.

Analyzing the data related to birth weight and premature birth, the study showed that there was no statistically significant association between birth weight, prematurity, and DDE. Similar results were reported by Allazzam [25], in contrast with Elfrink’s study, which showed that there is a lower risk for normal birth weight children to develop DDE in the primary dentition compared to children with low birth weight [27]. Other studies have also reported a higher risk of developing DDE in children born prematurely compared to those born at term [26,28,29]. The explanation could be that low birth weight may be associated with other possible causes of DDE, regarding the health status of the mother during pregnancy and possible complications during delivery. Thus, many premature babies require neonatal intensive care because of the physiological immaturity and health problems associated with premature birth, which also affect the function of ameloblasts in amelogenesis.

Our study did not reveal any statistically significant differences between the DDE group and the non-DDE group in breastfeeding or formula feeding of infants, or in breastfeeding duration. Similar results were reported by Vargas-Ferreira in 2018 [13] and Allazzam [25], while Koruyucu found a significant association between the average breastfeeding duration and DDE [26].

Another study conducted by Massoni in 2009 reported that the lack of breastfeeding constituted for those children a 3 times higher risk of DDE, compared to those who were breastfed [21]. The most pertinent explanation lies in the fact that the nutritional and immunological properties of milk are very important in ensuring the metabolic needs of the infant’s growth and development, including the formation of the dental organ [30]. However, a study conducted in Finland in 1996 reported that prolonged breastfeeding poses a risk for healthy infants to develop hypomineralization, possibly due to environmental contaminants found in breast milk [31].

The analysis of the children’s health status revealed that there were no statistically significant differences between the group with DDE and the group without DDE. The risk factors that can affect odontogenesis in the first years of life have been addressed in several studies [25,26,29,32,33,34]. Postnatal factors that may contribute to enamel hypoplasia include nutritional deficiencies, systemic conditions, and local trauma [33]. Malnutrition and ricketiness do not provide the ameloblasts with the necessary nutrients to secrete the enamel layer.

For children under 4, chickenpox (29.3%), repeated episodes of fever (26.1%), measles (14.7%), pneumonia (6.3%) and gastrointestinal conditions (3.9%) have been associated with DDE [29]. Allazzam also highlighted adenoid infections, fever, tonsillitis, asthma, and medical history of children, as etiological factors of DDE [25].

Koruyucu reported a significant association between diseases of the digestive system, with the presence of frequent episodes of diarrhea, asthma, repeated high fever, otitis, renal failure, rubella, varicella, parotitis and the occurrence of hypomineralization in the permanent dentition. At the same time, pneumonia, laryngeal and lower respiratory tract infections, urinary tract infections, rubella and scarlet fever were not considered risk factors for DDE [26].

Regarding medication administered in the first years of life, in the present study a significant association was identified between DDE and amoxicillin, ibuprofen, cephalosporins. There is no clear evidence to incriminate drugs as an etiological factor of DDE, as it is not clear whether they, or the conditions for which they are administered, are the true cause of DDE [26,35]. Animal studies revealed that amelogenesis can be influenced by the presence of high fever, exposure to dioxin and administration of amoxicillin. However, Elfrink disproved the hypothesis that amoxicillin administration in pregnancy could cause DDE in fetal primary teeth [27]. In contrast, in 2009, Laisi indicated that early administration of amoxicillin is one of the causes of DDE [36]. Allazzam also found a significant association between hypomineralization and frequent antibiotic administration [25].

The results from the present study overlap with those of Tapias and Simratvir. Thus, in 2001, Tapias found a direct relationship between the use of cephalosporins and the development of DDE in the permanent dentition [37], as did Simratvir, who reported in 2011 a case of generalized hypoplasia of permanent teeth, having as a possible cause long-term use of cephalosporins [5]. A study conducted by Laisi disproved the hypothesis that the presence of DDE would be the consequence of the use of cephalosporins [36].

Regarding the role of ibuprofen in the development of DDE, Serna Muñoz reported in 2018 results opposite to the present study, showing that the administration of the drug in the first years of life does not influence odontogenesis [38].

In Poiana Mare, the main occupation of the inhabitants is the cultivation of vegetables. Until the year 2000, chemical fertilizers based on ammonium nitrate were widely used for the maintenance of these crops, which was performed manually and mechanically. After 2000, in addition to chemical fertilizers, pesticides were also widely used to combat weeds and other pests. Further studies will show whether components of these substances are present in well water and whether their involvement in the development of DDE can be proven. There were no statistically significant differences between the DDE group and the non-DDE group in calcium deficiency, fruit/vegetable sources. Regarding water source, the study showed an association of DDE with private well water use. The rural area where the study was conducted is an agricultural area where chemicals are used to stimulate agricultural crops. We found no other studies associating well water with the development of DDE.

Calcium deficiency is recognized in the literature as an important risk factor for DDE. Thus, in 2013, Salanitri pointed out that vitamin D deficiency during pregnancy, which helps to fix calcium, is one of the prenatal risk factors that generate enamel hypoplasias [14]. Also, the hypoplasias found in children from indigenous communities may also be caused by insufficient intake and absorption of calcium and vitamin D [39].

We did not identify studies investigating the association between pesticide use and DDE occurrence, but in children with high lead levels, because of accidental exposure or ingestion of lead paint, hypoplasias with a characteristic appearance were reported in 1991 [23].

In addition to the data collected through the questionnaire, there are also risk factors that are harder to quantify, such as environmental ones. Thus, regarding the relationship between environmental factors and DDE, in 2009 Kuscu did not find a significant association between the presence of hypomineralization at the level of molars 1 and permanent incisors, and life in a polluted environment [40]. Thus, Kuscu reported a prevalence of defects of 9.10% for children who lived on a green energy island and 9.20% for children who lived in a highly industrialized area with a high degree of pollution. In contrast, Lukinmaa reported in 2001 a significant association between dioxin and DDE. Dioxin is a ubiquitous environmental pollutant, resulting mainly from combustion and as an unwanted by-product of various industrial activities. Thus, for healthy children, even exposure to a very low level of dioxin through breast milk may result in hypomineralization [41].

Hypomineralized teeth have a higher degree of porosity, which leads to lower mechanical strength and a high risk of carious damage [27]. It is a general impression that the presence of DDE makes the tooth enamel susceptible to carious processes, due to irregular and retentive surfaces, which lead to the accumulation of bacterial plaque and the higher acid solubility of the affected teeth [13]. The presence of an increased amount of plaque results in caries in both DDE and non-DDE teeth. Opydo-Szymaczeka reported in 2018 a significantly higher proportion of dental caries in children with DDE compared to the proportion of caries in children without DDE [42]. Americano also found in 2017, in a systematic review, an important connection between dental caries and DDE, children with dental defects having a 2–4 times higher risk than children in the control group, of developing carious lesions for all teeth [12].

The present research was carried out in a rural area in Romania, where access to education and health is lower. It is considered that the level of education can influence several non-economic aspects, such as behavior in society and health capital [43]. A high level of education is associated with better living conditions [44], and this is likely to influence tooth development [45].

The present study had some limitations. The group of children was small and consisted of children from a single rural locality, as the study only took place between January–February 2020, and was stopped in March 2020, after the debut of COVID-19 pandemics. Also, few of the children’s mothers omitted answers to one or more questions, which may have influenced the results. The subjective component in the mothers’ answers should be mentioned, as it is about events that took place in the past. Finally, the present study was retrospective, which did not allow the most effective determination of the relationship between cause and effect or the temporal relationship between DDE and carious lesions, in this sense prospective research would be more helpful.

## 5. Conclusions

This study emphasized an increased prevalence of DDE in children with rural residency from Dolj county.

It also showed that there is a significant association between the use of amoxicillin, ibuprofen, and cephalosporin during the period of formation of permanent teeth and the presence of DDE. Children who consumed water from private wells developed more enamel defects.

The presence of DDE was associated with a higher risk of caries development.

The dissemination of our results among mothers and physicians with pediatric specialties may help reduce the prevalence of this condition.

## Figures and Tables

**Table 1:** Modified DDE index.

	Normal	Demarcated Opacity	Diffuse Opacity	Hypoplasia	Other Defects
Index	0	1	2	3	4

**Table 2:** Statistical comparisons between DDE and no-DDE groups regarding parameters related to mothers’ status during pregnancy and newborns.

	Pregnancy (Mothers)–N (%)		Birth–N (%)	Newborns–N (%)	
	Comorbidities	Medication	Complications	Weight	Premature
DDE	1 (4.2)	6 (25)	1 (4.2)	-	3 (12.5)
No-DDE	5 (2.6)	21 (11.1)	10 (5.3)	-	13 (6.9)
χ^2^(1)/U	0.180	3.711	0.055	1760	0.969
*p*	0.516 *	0.094 *	0.985 *	0.559 **	0.400 *

* Fisher exact test (Chi-square test). ** Mann-Whitney U test.

**Table 3:** Statistical comparisons between DDE and no-DDE groups regarding parameters related to breastfeeding (expressed in months).

	Breastfeeding	
	Natural	Duration (Months)
χ^2^(1)/U	2.346	2252
*p*	0.228 *	0.955 **

* Fisher exact test. ** Mann-Whitney U test.

**Table 4:** Statistical comparisons between DDE and no-DDE groups regarding parameters related to conditions during the first years of life.

	First Years of Life			
	Chickenpox	Otitis	Pharyngitis	Other Conditions
DDE	3 (12.5%)	2 (8.3%)	2 (8.3%)	1 (4.2%)
No-DDE	36 (19.0%)	10 (5.3%)	20 (10.6%)	7 (3.7%)
χ^2^(1)/U	0.610	0.371	0.116	0.013
*p*	0.580 *	0.630 *	0.833 *	0.981 *

* Fisher exact test.

**Table 5:** Statistical comparisons between DDE and no-DDE groups regarding parameters related to medication administered—N (%).

	Amoxicillin	Penicillin	Ibuprofen	Paracetamol	Cephalosporins	Other Medication
DDE	15 (62.5)	2 (8.3)	5 (20.8)	1 (4.2)	4 (16.7)	1 (4.2)
No-DDE	75 (39.7)	21 (11.1)	12 (6.3)	14 (7.4)	8 (4.2)	10 (5.3)
χ^2^(1)	4.544	0.171	6.083	0.342	6.193	1.332
*p*	0.033 *	0.680 *	0.029 **	0.559 *	0.033 **	0.608 **

* Chi-square test. ** Fisher exact test. Bold indicates statistically significant results.

**Table 6:** Statistical comparisons between DDE and no-DDE groups regarding lack of calcium, sources of water and fruits/vegetables and use of pesticides—N (%).

	Lack of Calcium	Private Source		Use of Pesticides
		Water	Fruits/Vegetables	
DDE	4 (16.7)	23 (95.8)	8 (33.3)	2 (8.3)
No-DDE	44 (23.3)	145 (76.7)	53 (28.0)	28 (14.8)
χ^2^(1)	0.534	4.669	1.478	0.739
*p*	0.465 *	0.031 *	0.478 *	0.541 **

* Chi-square test. ** Fisher exact test. Bold indicates statistically significant results.

**Table 7:** Distribution of caries and DDE teeth.

	Primary Teeth—N (%)			Permanent Teeth—N (%)		
	Caries	No Caries	Total	Caries	No Caries	Total
DDE	4 (0.31)	2 (0.16)	6 (0.47)	8 (0.19)	35 (0.84)	43 (1.03)
No-DDE	260 (20.17)	1023 (79.36)	1283 (99.53)	221 (5.31)	3899 (93.66)	4120 (98.97)
Total	264 (20.48)	1025 (79.52)	1289 (100)	229 (5.50)	3934 (94.50)	4163 (100)

## Data Availability

The authors declare that the data of this research is available from the correspondence authors upon reasonable request.
